# Myeloid Differentiation Protein 2 Mediates Angiotensin II-Induced Liver Inflammation and Fibrosis in Mice

**DOI:** 10.3390/molecules25010025

**Published:** 2019-12-19

**Authors:** Yi Zhang, Hui Liu, Wenjing Jia, Jiayu Qi, Wentao Zhang, Wenxin Zhang, Guang Liang, Yali Zhang, Hongjin Chen

**Affiliations:** 1 School of Chemical Engineering, Nanjing University of Science and Technology, Nanjing 210094, China; zhangy@wmu.edu.cn (Y.Z.); wzmcliangguang@163.com (G.L.); 2 Chemical Biology Research Center, School of Pharmaceutical Sciences, Wenzhou Medical University, Wenzhou 325035, China; liuhui_bio@163.com (H.L.); 15067897058@163.com (W.J.); 15067825567@163.com (J.Q.); moxiaozjun@outlook.com (W.Z.); magicicizhang0127@163.com (W.Z.); ya-li000@163.com (Y.Z.)

**Keywords:** liver injury, Angiotensin II, myeloid differentiation 2, L6H21, inflammation

## Abstract

Angiotensin II (Ang II) participates in the pathogenesis of liver injury. Our previous publications reported that myeloid differentiation protein 2 (MD2) mediates Ang II-induced cardiac and kidney inflammation by directly binding to Ang II. Thus, we hypothesize that MD2 is critical to Ang II-induced liver injury. Subcutaneous injections of Ang II for 8 weeks were adopted to build the liver injury model. With a specific MD2 inhibitor L6H21 and MD2 knockout mice, we reported that MD2 inhibition and knockout significantly mitigate liver inflammation and fibrosis in mice injected with Ang II. To be more specific, the functional and pathological damages induced by Ang II were mitigated by L6H21 or MD2 knockout. MD2 knockout or L6H21 administration inhibited the Ang II-induced upregulation of fibrosis markers, inflammatory cytokines, and adhesion molecules in gene or protein levels. The activation of NF-κB and Extracellular signal-regulated kinases (ERK) induced by Ang II was also reversed by L6H21 treatment or MD2 deficiency. Note that the co-immunoprecipitation study showed that L6H21 downregulated the ANG II-induced toll-like receptor 4 (TLR4)/MD2 complex in liver tissues while having no effects on MD2 expression. Our results reported the critical role of MD2 in the progress of liver injury and suggested that MD2 is a potential therapeutic target for liver injury.

## 1. Introduction

Liver injury-correlated diseases are among the commonest health problems, with almost 800,000 deaths each year worldwide [1]. Continuous liver injury induces hepatic inflammation and fibrosis, thus resulting in cirrhosis, liver failure, or hepatocellular carcinoma. Numerous factors, covering high-fat diets, alcohol, carbon tetrachloride, drugs, ischemia, and reperfusion, can induce liver injury [2]. Recently, clinical and experimental studies have progressively highlighted the renin–angiotensin system (RAS) in hepatic diseases [3,4]. Angiotensin (Ang) II, the major RAS component, has been shown to regulate inflammation and fibrosis in various diseases (e.g., liver injury) [3,5]. Prolonged infusion of Ang II into normal rats can induce stellate cell activation and proinflammatory effects in the liver, which is independent of the rise in arterial pressure [6]. Nevertheless, whether there is a critical protein participating in the process remains unclear.

Myeloid differentiation 2 (MD2), an accessory protein of toll-like receptor 4 (TLR4), is vital to mediating the lipopolysaccharide (LPS)/TLR4 acute inflammatory signaling [7,8]. Increasing evidence has shown that MD2 is associated with numerous acute and chronic inflammatory responses, and is considered a potential and meaningful therapeutic target for several inflammatory diseases [9,10,11]. Hosoki reported that MD2 plays an important role in the induction of allergic sensitization to cat dander and common pollens relevant to human allergic diseases [12]. Others and our group have reported that MD2 participates in the development of non-alcoholic fatty liver disease, as well as methionine- and choline-deficient diet-induced steatosis and fibrosis [13,14]. Recently, our group investigated whether MD2 mediates Ang II-induced cardiac and kidney inflammation by directly binding to Ang II and activating the TLR4/ Myeloid differentiation primary response 88 (MyD88) signaling pathway, which is independent of the AT1 receptor [15,16]. Furthermore, L6H21, an inhibitor of MD2, protects against Ang II-induced cardiac and kidney injury both in vitro and in vivo.

In the present study, we tested the effectiveness of MD2, using the small molecular inhibitor of MD2, L6H21 [17], and MD2 knockout mice, in reducing inflammation and fibrosis levels in an Ang II-induced mouse model of liver injury. According to the results, MD2 inhibition with L6H21 was as feasible as MD2 knockout in inhibiting the development of inflammatory damage and fibrosis in liver injury. L6H21 significantly reduced the complex of the MD2/TLR4 axis in liver tissues. We concluded that MD2 is a potentially crucial component of inflammation and fibrosis in ANG II-induced liver injury.

## 2. Results

### 2.1. Ang II Increases Liver MD2 Expression

**Figure 1:**
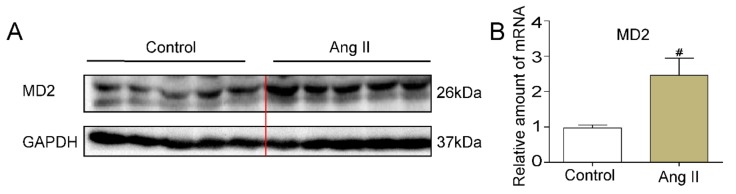
Liver myeloid differentiation protein 2 (MD2) expression was upregulated by Angiotensin (Ang) II. C57BL/6 mice were injected with Ang II (1.4 mg/kg/day in phosphate buffer, pH 7.2) or PBS (Control) for 8 weeks (*n* = 7 in each group). Liver tissues were harvested. (**A**) MD2 expression was ascertained by Western blot (IB) analysis (representative of five independent determinations). (**B**) ANG II increased mouse liver mRNA levels of MD2. Real-time qPCR assay was used to examine the mRNA expression of MD2. The mRNA values were normalized to the housekeeping gene β-actin and reported as mean ± SEM (*n* ≥ 5, ^#^ *p* < 0.05, vs. Control group).

A mouse liver injury model was built by injections of Ang II (1.4 mg/kg/day in a phosphate buffer, pH 7.2) continued for 8 weeks, and the MD2 protein level in liver tissue was upregulated (Figure 1A). The mRNA level of MD2 also significantly increased in the ANG II group compared with the control group (Figure 1B). These results revealed that MD2 might participate in Ang II-induced liver injury.

### 2.2. MD2 Inhibition and Knockout Protected Mice from Ang II-Induced Liver Injury and Dysfunction

**Figure 2:**
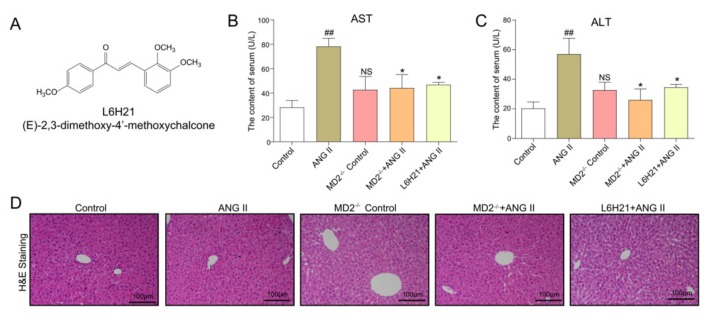
MD2 inhibition or MD2 knockout protected mice from Ang II-induced liver injury and dysfunction. (**A**) The structure of L6H21. (**B,C**) Liver function was ascertained by measuring (**B**) aspartate aminotransferase (AST) and (**C**) serum alanine aminotransferase (ALT) level in serum. (**D**) Representative histopathological variations in liver tissue ascertained with hematoxylin and eosin (H&E) staining (images captured at 200× magnification). Data are expressed as mean ± SEM (*n* ≥ 5, ^##^ *p* < 0.01, vs. Control group; NS, no significant difference vs. Control group; * *p* < 0.05, vs. Ang II group).

To assess the implication of MD2 in Ang II-induced liver injury in vivo, MD2 knockout mice and the MD2 inhibitor L6H21 (Figure 2A) were adopted for study. Serum alanine aminotransferase (ALT) and aspartate aminotransferase (AST) refer to two of the hallmarks of liver functional injury. Compared with the control mice, Ang II upregulated both AST (Figure 2B) and ALT (Figure 2C) levels in serum. Nevertheless, the administration of L6H21 or MD2 knockout downregulated the biochemical disorders induced by Ang II. Figure 2D suggests that the hematoxylin and eosin (H&E) staining exhibited normal liver architecture in control mice and structural abnormalities in the Ang II-treated mice, while the Ang II-induced structural abnormalities were mitigated in MD2 knockout mice and those administered with 5 mg/kg L6H21. According to these results, MD2 inhibition and knockout can protect mice from Ang II-induced liver injury and dysfunction.

### 2.3. MD2 Inhibition and Knockout Protected Mice from Ang II-Induced Liver Fibrosis

**Figure 3:**
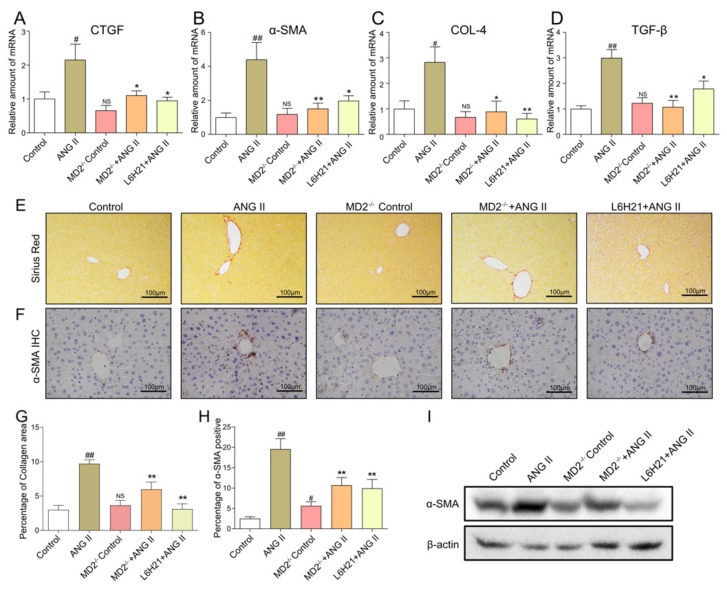
MD2 inhibition or MD2 knockout protected mice from Ang II-induced liver fibrosis. Mouse liver samples were prepared as described in materials and methods. (**A**–**D**) The mRNA levels of CTGF (**A**), α-SMA (**B**), COL-4 (**C**), and TGF-β (**D**) in liver tissues were ascertained by real-time qPCR. Representative light micrographs of the histochemical assessment of liver tissues: Sirius Red staining (**E**) and α-SMA immunohistochemistry (**F**) were employed for the detection of fibrosis (images captured at 200× magnification). (**G**) Quantification of the collagen area in panel E. (**H**) Quantification of the α-SMA-positive area in panel F. (**I**) The protein level of α-SMA in liver tissue was ascertained by Western blot. Data are presented as mean ± SEM (*n* ≥ 5, ^#^ *p* < 0.05, ^##^ *p* < 0.01, vs. Control group; NS, no significant difference, vs. Control group; * *p* < 0.05, ** *p* < 0.01, vs. Ang II group).

Next, the effects of MD2 on Ang II-induced liver fibrosis were assessed. Real-time qPCR assay revealed that hepatic fibrosis-related markers, CTGF, α-SMA, COL-4, and TGF-β, were upregulated in Ang II-treated mice, whereas they were downregulated in Ang II-treated MD2 knockout mice and Ang II-treated mice administered with 5 mg/kg L6H21 (Figure 3A–D). Sirius Red staining (Figure 3E,G) was also employed to ascertain the effect of MD2 on Ang II-induced liver fibrosis. The results suggested that Ang II significantly induced collagen deposition in the liver, while both MD2 inhibition and knockout reduced Ang II-induced fibrosis. In the meantime, the fibrosis marker α-SMA was studied by immunohistochemistry staining (Figure 3F,H) and Western blot (Figure 3I) assay. The results suggested that Ang II treatment significantly upregulated α-SMA expression in liver, and MD2 inhibition and knockout reduced it. These observations revealed that MD2 in the liver is associated with fibrosis.

### 2.4. MD2 Inhibition and MD2 Knockout Protected Mice from Ang II-Induced Liver Inflammation

**Figure 4:**
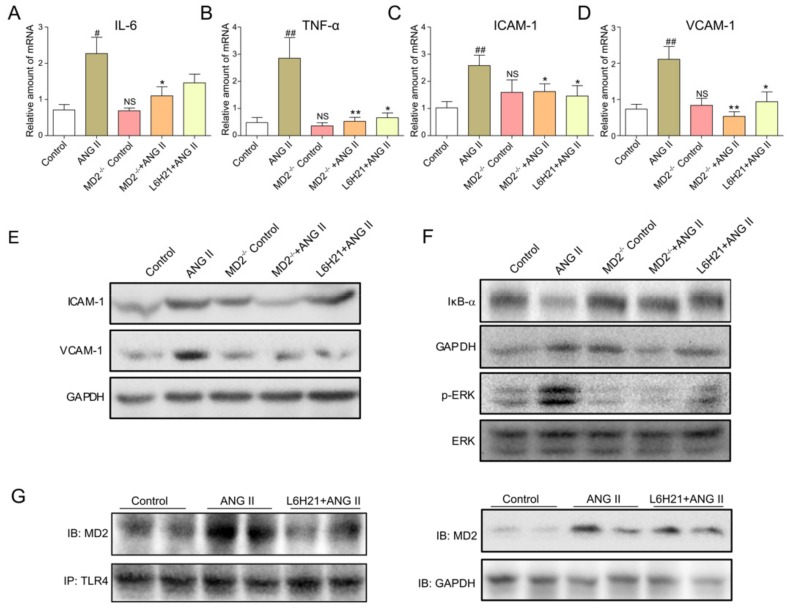
MD2 inhibition or MD2 knockout protected mice from Ang II-induced liver inflammation. Mouse liver samples were prepared as described in Materials and Methods. (**A**–**D**) The mRNA levels of IL-6 (**A**), TNF-α (**B**), VCAM-1 (**C**), and ICAM-1 (**D**) in liver tissues were ascertained by real-time qPCR. Data are presented as mean ± SEM (*n* ≥ 5, ^#^ *p* < 0.05, ^##^ *p* < 0.01, vs. Control group; NS, no significant difference vs. Control group; * *p* < 0.05, ** *p* < 0.01, vs. Ang II group). (**E**) The protein levels of ICAM-1 and VCAM-1 in liver tissues were ascertained by Western blot. (**F**) IκB-α degradation and ERK phosphorylation were ascertained by Western blot. (**G**) The complex of MD2/toll-like receptor 4 (TLR4) and input MD2 expression in liver tissue were determined by immunoprecipitation and Western blot, respectively.

The variations of proinflammatory cytokines (IL-6 and TNF-α) and adhesion molecules (ICAM-1 and VCAM-1) in mRNA levels in liver tissue were ascertained by real-time qPCR. As shown in Figure 4A–D, L6H21 treatment and MD2 deficiency effectively reduced the transcription of IL-6 (Figure 4A), TNF-α (Figure 4B), ICAM-1 (Figure 4C), and VCAM-1 (Figure 4D) in the liver tissue of Ang II-treated mice. The protein level of ICAM-1 and VCAM-1 were also increased in the livers of Ang II-treated mice, while these changes were attenuated by MD2 inhibition or knockout (Figure 4E). To investigate the potential mechanism of L6H21 or MD2 deficiency on Ang II-induced inflammation, ERK and NF-κB signaling were studied. The results showed that L6H21 treatment or MD2 knockout significantly inhibited the Ang II-induced IκB-α degradation and ERK phosphorylation in liver tissue (Figure 4F). Since L6H21 acts as an inhibitor of MD2, immunoprecipitation assay was adopted to ascertain the effects of L6H21 on Ang II-induced TLR4/MD2 complex formation in liver. Figure 4G suggests that L6H21 markedly reduced Ang II-induced formation of TLR4/MD2, while it had no effects on Ang II-induced MD2 expression. Thus, these results indicated that MD2 blockage remarkably inhibited liver inflammation, and also inhibited the activation of NF-κB and ERK induced by Ang II in liver tissue, which may be associated with the liver protective effect.

## 3. Discussion

There is accumulating evidence to show that the RAS participates in the development of hepatic diseases [3]. Activation of RAS is a common finding in advanced cirrhosis. A reason for this may be a systemic hemodynamic derangement, presented as splanchnic and peripheral excessive vasodilation, leading to reactive secretion of vasoconstrictors and “underfilling” in the central intrathoracic compartment [18]. Ang II, a crucial vasoconstrictive peptide of the RAS, induces inflammatory cytokine expression and stimulates fibrogenesis [19]. The model of continuous infusion of Ang II has been extensively used in other organs (e.g., the kidney and the heart). In the present study, 8 weeks of Ang II infusion resulted in significant upregulation of serum ALT and AST, indicative of liver dysfunction. The liver dysfunction was accompanied by light and electron microscopic morphological evidence of liver injury, as well as a rise in several indicators of liver inflammation and fibrosis. These results agree with the report by Moreno [20] and Bataller [6], who used Ang II infusion with a rat model, and concluded that Ang II continuous infusion can significantly induce liver inflammation and fibrosis.

MD2, a critical protein in the recognition of LPS by TLR4, has been reported to be vital to the development of liver diseases. In NAFLD models induced by both a methionine- and choline-deficient and high-fat diet, MD2 knockout significantly mitigated liver steatosis, steatohepatitis, and fibrosis [13,14]. Baicalein, an inhibitor of MD2, has been reported to protect against LPS-induced liver injury by targeting MD2 in mice [21]. Our results suggested that MD2 deficiency and inhibition by L6H21 mitigate Ang II-induced liver inflammation, fibrosis, and injury. In the previously reported evidence, cell-free assays showed direct binding of Ang II with MD2 [15,16]. This direct interaction of Ang II with MD2 was similar to LPS binding with MD2, resulting in a MD2/TLR4 complex formation, as well as the recruitment of adaptor molecules for signaling. This study showed that MD2 inhibition or knockout attenuated Ang II-induced NF-κB activation and phosphorylation of ERK in liver, which was also found in heart and kidney tissue [15,16]. These results indicated that the inhibitor of L6H21 or MD2 knockout attenuated Ang II-induced inflammation in liver tissue by inhibiting NF-κB and ERK pathways.

## 4. Materials and Methods

### 4.1. Reagents

Ang II was provided by Sigma (St. Louis, MO, USA). MD2 (no. ab24182), TLR4 (no. ab22048), α-SMA (no. ab5694), ICAM-1 (no. ab171123), and VCAM-1 (no. ab134047) antibodies were purchased from Abcam (Cambridge, MA, USA). GAPDH (no. sc-293335) and β-actin (sc-47778) antibodies were obtained from Santa Cruz Technology (Santa Cruz, CA, USA). ERK (9101S), *p*-ERK (9102S), and IκB-α (4812S) were purchased from Cell Signaling Technology (Danvers, MA, USA). ALT and AST detection kits were purchased from Nanjing Jiancheng Bioengineering Institute (Nanjing, China). L6H21 was synthesized and structurally identified using MS and ^1^H NMR analyses, as described in our previous report [22]. For in vivo studies, L6H21 was dissolved in 1% CMC-Na, and 1% CMC-Na also acted as the vehicle control.

### 4.2. Animals

The six-week-old males C57BL/6 (WT) weighing 16–18 g were provided by the Animal Centre of Wenzhou Medical University (Wenzhou, China). Fourteen male MD2^−/−^ mice with C57BL/6 background (B6.129P2-Ly96 KO) were introduced from the Riken Bio Resource Center of Japan (Tsukuba, Japan). Mice were housed at 22 °C with a 12:12 h light–dark cycle and fed with a standard rodent diet, as well as free access to water. The mice were acclimated to the laboratory for at least two weeks before being employed for study. All animal care and experimental procedures in accordance with the “Detailed Rules and Regulations of Medical Animal Experiments Administration and Implementation” (Order No. 1998–55, Ministry of Public Health, China) were approved by the Wenzhou Medical University Animal Policy and Welfare Committee. Protocols involving the use of animals were approved by the Wenzhou Medical University Animal Policy and Welfare Committee. Liver fibrosis was induced in mice by daily subcutaneous injections of Ang II (1.4 mg/kg/day in phosphate buffer, pH 7.2) for eight weeks. The mice were randomly split into five groups with eight mice in each group: (1) control group (WT + PBS); (2) WT + Ang II; (3) MD2^−/−^ + PBS; (4) MD2^−/−^ + Ang II; and (5) Ang II + L6H21. After eight weeks of treatment, the mice were sacrificed with ether anesthesia, and the blood and liver samples were harvested for analyses as described below.

### 4.3. Histopathologic Analysis

For routine histology, liver tissues were harvested and fixed in 4% paraformaldehyde for 24 h, followed by post-fixation with 70% ethanol. Subsequently, tissues were processed into paraffin blocks, sectioned at 5 μm, and then stained by H&E (Solarbio, Beijing, China) and Sirius Red staining. The sections were observed under a light microscope (200× magnification; Nikon, Tokyo, Japan).

### 4.4. Immunohistochemistry

The 5 μm liver sections were subjected to deparaffinization and rehydration followed by treatment with 3% H_2_O_2_ for 10 min. After being incubated with 1% BSA in a phosphate buffer for 30 min, the liver sections were incubated with primary antibodies of α-SMA (1:200) overnight at 4 °C. Subsequently, the tissues were incubated with a 1:500 dilution of HRP-conjugated secondary antibody for 1 h at ambient temperature. The nuclei were counter-stained with hematoxylin for 1 min. These sections were observed under a light microscope (200× magnification; Nikon, Tokyo, Japan). 

### 4.5. Measurement of ALT and AST in Serum

The serum levels of ALT and AST were ascertained using commercial kits (Nanjing Jiancheng Bioengineering Institute, Nanjing, China) following the manufacturer’s instructions.

### 4.6. Western Blot Analysis

Liver tissue lysate was prepared and protein concentration was ascertained by Coomassie Blue staining assay (Beyotime, Shanghai, China). Proteins were separated using 10% sodium dodecyl sulfate-polyacrylamide gel electrophoresis and then transferred onto polyvinyldene fluoride membrane (Bio-Rad, Hercules, CA, USA). Membranes were blocked with 5% milk in tris-buffered saline, supplemented by 0.05% Tween-20, for 1.5 h at ambient temperature. Subsequently, primary antibody incubation was carried out overnight at 4 °C, followed by HRP-conjugated secondary antibodies (1:10,000) for 1 h at ambient temperature. The immunoreactive proteins were visualized with a chemiluminescence reagent (Bio-Rad, Hercules, CA, USA).

### 4.7. Co-Immunoprecipitation

Nearly 600 μg of extracted proteins from liver tissues were adopted to achieve immunoprecipitation by the incubation of 3 μg of TLR4 antibodies overnight at 4 °C, and then precipitated with protein G-Sepharose beads at 4 °C for 2 h. The immunoprecipitates were washed five times with 1 mL cold PBS. Supernatants were separated and subjected to immunoblotting. They were separated on a 10% SDS-PAGE, followed by immunoblotting.

### 4.8. Reverse Transcription and Real-Time Quantitative PCR

Total RNA was isolated from liver tissues (20 mg) with TRIZOL (Invitrogen, Carlsbad, CA, USA). Reverse transcription assay was performed using an M-MLV kit (Invitrogen, Carlsbad, CA, USA). Real-time quantitative PCR was conducted using the Eppendorf Realplex4 instrument (Eppendorf, Hamburg, Germany). Primers of genes, TNF-α, IL-6, VCAM-1, ICAM-1, COL-4, CTGF, α-SMA, TGF-β, and β-actin were synthesized from Invitrogen (Invitrogen). The relative amount of each gene was normalized to the amount of β-actin. The primer sequences applied are shown as follows:

Mouse TNF-α forward primer: 5′-TGATCCGCGACGTGGAA-3′;

Mouse TNF-α reverse primer: 5′-ACCGCCTGGAGTTCTGGAA-3′.

Mouse IL-6 forward primer: 5′-GAGGATACCACTCCCAACAGACC-3′;

Mouse IL-6 reverse primer: 5′-AAGTGCATCATCGTTGTTCATACA-3′.

Mouse VCAM-1 forward primer: 5′-TGCCGAGCTAAATTACACATTG-3′;

Mouse VCAM-1 reverse primer: 5′-CCTTGTGGAGGGATGTACAGA-3′.

Mouse ICAM-1 forward primer: 5′-GCCTTGGTAGAGGTGACTGAG-3′;

Mouse ICAM-1 reverse primer: 5′-GACCGGAGCTGAAAAGTTGTA-3′.

Mouse CTGF forward primer: 5′-ACTATGATGCGAGCCAACTGC-3′;

Mouse CTGF reverse primer: 5′-TGTCCGGATGCACTTTTTGC-3′.

Mouse TGF-β forward primer: 5′-TGACGTCACTGGAGTTGTACGG-3′;

Mouse TGF-β reverse primer: 5′-GGTTCATGTCATGGATGGTGC-3′.

Mouse α-SMA forward primer: 5′-GTCCCAGACATCAGGGAGTAA-3′;

Mouse α-SMA reverse primer: 5′-TCGGATACTTCAGCGTCAGGA-3′.

Mouse COL-4 forward primer: 5′-GCTCCACCACTCAAAGGTGTT-3′;

Mouse COL-4 reverse primer: 5′-GGCACAGTCGAGTCTTCCA-3′.

Mouse β-Actin forward primer: 5′-CCGTGAAAAGATGACCCAGA-3′;

Mouse β-Actin reverse primer: 5′-TACGACCAGAGGCATACAG-3′.

### 4.9. Statistical Analysis

The statistical significance of differences between groups was calculated by the Student’s-test between two groups, or one-way ANOVA in more than two groups, with GraphPad Pro 5.0 (GraphPad, San Diego, CA, USA). Results are expressed as mean ± SEM. Differences were considered to be of significance at ^#^ *p* < 0.05, ^##^ *p* < 0.01 vs. Control group; * *p* < 0.05, ** *p* < 0.01 vs. Ang II group.

## 5. Conclusions

In conclusion, our study suggested that MD2 deficiency or inhibition by L6H21 can mitigate Ang II-induced liver dysfunction, pathological injury, inflammation and fibrosis, and activation of NF-κB and ERK pathways through reducing the formation of the MD2/TLR4 complex. Our results conclusively showed that MD2 participates in Ang II-induced liver injury development. Furthermore, MD2 inhibition may offer a promising target to inhibit liver inflammation and fibrosis.
